# Relationaler Umgang mit Krisen von Jugendamtsfachkräften in der Fallarbeit hinsichtlich der Auswirkungen der Covid-19-Pandemie

**DOI:** 10.1007/s11618-023-01143-1

**Published:** 2023-02-10

**Authors:** Pascal Bastian, Megan Benoit, Katharina Freres, Jana Posmek

**Affiliations:** Fachbereich Erziehungswissenschaften, Institut für Bildung im Kindes- und Jugendalter, Arbeitsbereich Sozialpädagogik, Rheinland-Pfälzische Technische Universität Kaiserslautern Landau, Bürgerstr. 23, 76829 Landau, Deutschland

**Keywords:** Krise, Umgang mit Krisen, Covid-19-Pandemie, Kinderschutz, Crisis, Dealing with crises, Covid-19-pandemic, Child protection

## Abstract

Über die Auswirkungen der Covid-19 Pandemie auf die Fallarbeit der Jugendämter liegt bislang noch wenig empirisches Wissen vor. Allerdings geben qualitative Studien eine Reihe von Hinweisen zur Bedeutung von Teamarbeit, zu expliziten und impliziten Handlungsweisen sowie zu Entscheidungspraktiken (*Sense-Making*) von Fachkräften im Kinderschutz. Diese Aspekte wurden durch die Lockdowns und die pandemiebedingten Schutzmaßnahmen weitgehend eingeschränkt. Daraus ergibt sich die Frage der vorliegenden Studie nach dem Umgang mit diesen Einschränkungen und den darauf bezogenen Umgang.

Grundlage der Analyse bildet die kontrastive Auswertung von Telefoninterviews mit Fachkräften zweier Jugendämter. Aus einer relationalen Perspektive im Sinne Bruno Latours, die den Blick nicht alleine auf die jeweiligen Akteur*innen, sondern vielmehr auf deren Verbindungen und Vernetzungen untereinander richtet, werden zunächst Verschiebungen des Netzwerkes, in dem die Fälle üblicherweise bearbeitet werden, sichtbar. Diese Transformationen wurden auf der Grundlage der Interviews als krisenhaft erlebte Einschränkungen der üblichen Handlungspraxis herausgearbeitet. In den analysierten Daten lassen sich Handlungsweisen rekonstruieren, die sich als Umgang mit dieser Krise fassen lassen. Die Ergebnisse zeigen, dass die eigentliche Krise nicht in der abstrakten Vorstellung einer Viruspandemie, sondern vor allem im Wegbrechen von Fallarbeitsgewohnheiten zu finden ist.

## Abstract (en)

There is still little empirical knowledge about the impact of the Covid 19 pandemic on the casework of youth welfare offices. However, qualitative studies provide a number of indications on the importance of teamwork, on explicit and implicit ways of acting as well as on decision-making practices (sense-making) of professionals in child protection. These aspects were largely limited by the lockdowns and the pandemic-related protective measures. This leads to the question of the present study about how these restrictions are dealt with and the coping strategies related to them.

The analysis is based on the contrastive interpretation of telephone interviews with professionals from two youth welfare offices. From a relational perspective in the sense of Bruno Latour, which does not focus on the actors, but rather on their connections and relations, some transformations in the network in which the cases are usually processed become visible. These transformations were elaborated based on the interviews as crisis-like restrictions of the usual practice of action. In the analyzed data, ways of acting can be reconstructed that can be understood as dealing with these crises. The results show that the actual crisis is not to be found in the abstract idea of a virus pandemic, but above all in the breaking away of case work habits.

## Einleitung

Der weltweit zu beobachtende politische Umgang mit Covid-19 und die damit verbundenen erheblichen Einschränkungen des Alltagslebens zur Verhinderung der Überlastung von Gesundheitssystemen zeigen sich als ein temporärer Ausnahmezustand (Scherr 2020). Grenzschließungen, Kontaktverbote, Schließung von Sozial- und Bildungseinrichtungen, sowie die einschneidenden Maßnahmen bezüglich des sozialen Miteinanders stellen ein bislang global einmaliges und beispielloses Ereignis dar, für das in keinem der betroffenen Systeme eine wirkliche erfahrungs- oder gar evidenzbasierte Umgangsweise vorliegt. Hiervon sind ebenfalls die Kinder- und Jugendhilfe sowie der Kinderschutz betroffen; ein Feld, dessen Bedeutung insbesondere in solchen Krisenzeiten mittlerweile medial vermehrt diskutiert und von Expert*innen aus Wissenschaft und Praxis betont wird.

Obwohl der staatliche Umgang mit Kindesmisshandlung, -vernachlässigung und -missbrauch in den letzten Jahren zunehmend im Fokus von Öffentlichkeit und Wissenschaft stand, lässt sich insgesamt noch von einem Forschungsbereich mit vielen Desideraten sprechen. Vor allem existiert noch (zu) wenig Wissen darüber, wie Fachkräfte der öffentlichen Kinderschutzbehörden zu den teilweise weitreichenden Urteilen und Entscheidungen kommen. Erst in jüngster Zeit finden sich eine Reihe qualitativer und vor allem ethnografischer Studien, die Auskunft über die konkrete Handlungspraxis der Fachkräfte geben (z. B. Ackermann 2017; Bastian 2017; Bastian et al. 2017; Cook 2019; Ferguson 2017). Diese lassen den Schluss zu, dass der Face-to-Face-Interaktion bei der Verdachtsarbeit etwa durch Hausbesuche, der Beratung von Eltern und Kindern sowie dem kollegialen Austausch im Team eine hohe Relevanz beigemessen wird. Aufgrund der Coronapandemie kann dieser hohen Bedeutungszuschreibung jedoch nicht nachgekommen werden, da der Virus jene Face-to-Face-Kontakte sowie die Teamarbeit erschwert bzw. teilweise verunmöglicht.

Im Folgenden stellen wir erste explorative Ergebnisse einer an den Stand der Forschung zu Handlungs- und Entscheidungspraxen im Rahmen des Kinderschutzes anknüpfenden Interviewstudie über die Fallarbeit in der Pandemie dar. Der Fokus des Artikels liegt dabei auf einem kontrastiven Fallvergleich von vier Interviews innerhalb zweier Behörden und konzentriert sich auf den Umgang mit dem krisenhaften Wegbrechen eingespielter Fallarbeitsgewohnheiten aufgrund der Covid-19-Pandemie.

Hierfür werden wir einen Überblick der bisherigen noch überschaubaren Forschungsveröffentlichungen zu Covid-19 im Bereich der Sozialen Arbeit aufzeigen, um anschließend den Blick auf den Umgang mit Krisen zu richten. Einen relationalen Forschungszugang auf der Basis der Arbeiten von Bruno Latour zugrunde legend, diskutieren wir Ergebnisse bisheriger Forschung zu Handlungsweisen im Kinderschutz, die als *Gewohnheiten* (Latour 2014) erfassbar sind. Nachdem wir das methodische Vorgehen erläutert haben, werden wir auf dieser Grundlage mit Verweisen auf unser empirisches Material die durch die Corona-Bestimmungen und des Lockdowns vollzogene Transformation des Netzwerks, in dem sich das Kinderschutzhandeln entfaltet, veranschaulichen. Hieran schließt die Darstellung sowie Diskussion der damit zusammenhängenden und sich aus der Interviewanalyse ergebenen Strategien des Umgangs mit der Krise an. Wir schließen mit einem Plädoyer für mehr ethnografische Forschung, um Praktiken und somit konkrete Prozesse des Umgangs mit Krisen aus einer relationalen Perspektive sichtbar zu machen.

## Forschungsstand

### Auswirkungen des Lockdowns während der Covid-19 Pandemie auf die Kinderschutzarbeit

Der bisherige Forschungsstand zu den Auswirkungen der Pandemie auf die Kinder- und Jugendhilfe ist noch überschaubar. Zur Fallpraxis im ASD gibt es mittlerweile allerdings einige publizierte Ergebnisse, welche Hinweise auf die Auswirkungen der Krise geben. Dabei lassen sich insbesondere zu den ***Konsequenzen und Herausforderungen für die Arbeit mit Familien***, die aus dem Wegbrechen von Unterstützungssystemen sowie dem „Social Distancing“ resultierten, bereits einige Daten finden. Im ASD ist eine Reduktion der Vis-a-Vis Kontakte mit Adressat*innen zu beobachten bzw. der Versuch, direkte Interaktionen vermehrt durch digitale Formaten zu substituieren (Baginsky und Manthorpe 2021; Cook und Zschomler 2020), wodurch das Begleiten von Menschen in Krisensituationen als herausfordernd erlebt wurde (Buschle und Meyer 2020; Meyer und Alsago 2021). Zugleich zeigte sich aber auch, dass viele Jugendämter die alltägliche Hilfe durch Flexibilisierung und Digitalisierung erfolgreich weiterführen konnten (D: Gerber und Jentsch 2021; Jentsch und Gerber 2022; UK: Ferguson et al. 2021; USA: Kothari et al. 2022). Nichtsdestotrotz wurden weiterhin aufgrund fehlender technischer Möglichkeiten in großem Umfang Hausbesuche durchgeführt (Mairhofer et al. 2020).

Daneben betreffen die Maßnahmen auch die ***Teamarbeit ***der Fachkräfte. Der kollegiale Austausch konnte nicht mehr wie bisher erfolgen, da vor allem zu Beginn des Lockdowns viele Sozialarbeiter*innen im Homeoffice gearbeitet haben (D: Mairhofer et al. 2020; UK: Cook und Zschomler 2020). Diese Verringerung des kollegialen Austauschs wird nicht nur negativ bewertet; einige Fachkräfte sprechen sogar von einer Intensivierung des Austausches durch digitale Formate (Buschle und Meyer 2020). Vor allem durch eine gute Betreuung seitens der Führungskräfte und eigene Stressbewältigungs- und andere Selbstfürsorgepraktiken der Fachkräfte konnten Belastungen und Ängste während der COVID-19-Pandemie besser bewältigt werden (Aughterson et al. 2021; Karadzhov et al. 2022).

Schließlich hat die Pandemie aber auch Auswirkungen auf die Zusammenarbeit mit dem Melder*innennetzwerk und den freien Trägern, d. h. auf das Gesamtnetzwerk, in dem Kinder- und Jugendhilfe sich vollzieht. Aufgrund der Schließungen von etablierten Hilfe- und Kontrollnetzwerken wie Kindertageseinrichtungen, Schulen oder Jugendzentren, gelangten insgesamt weniger Informationen und Meldungen an die Jugendämter (D: Mairhofer et al. 2020; UK: Cook und Zschomler 2020; USA: Brown et al. 2022). Einige Jugendämter berichten in der Studie des DJI jedoch, dass kompensatorisch die Meldungen von Nachbar*innen, der Polizei und von Kindern und Jugendlichen selbst zugenommen haben (Mairhofer et al. 2020).

### Forschungen zur konkreten Urteilspraxis im Kinderschutz

Wenn auch der Forschungsstand zur Urteilsbildung und Entscheidungsfindung während der Pandemie noch überschaubar ist, wurde dazu allgemein in der Sozialen Arbeit bzw. im Kinderschutz in den letzten Jahren verstärkt international geforscht (Zum Überblick: McCafferty und Taylor 2020; Taylor und Whittaker 2018). Wie bereits in unseren früheren Studien ausgearbeitet (Bastian und Schrödter 2014), liegt der Schwerpunkt dabei auf statistischen Studien, die ausgehend von normativen Annahmen zur professionellen Entscheidungsfindung (etwa im Sinne des „Heuristics and Biases“-Ansatzes von Tversky und Kahneman 1974) die Effektivität der Urteilspraxis untersuchen (Baird und Wagner 2000; Johnson et al. 2015; van der Put et al. 2017). Es besteht weiterhin ein Defizit an Studien, welche die Prozesse in der professionellen Alltagspraxis in den Blick nehmen. Dennoch lässt sich auch dazu gerade in den letzten Jahren ein leichter Anstieg konstatieren, vor allem durch *ethnographische Studien*, sei es bei Hausbesuchen (Bastian et al. 2017; Cook 2020; Ferguson 2017; Verhallen et al. 2017) oder im Jugendamt (Ackermann 2017; Büchner 2014; Freres et al. 2019; Leigh 2017; Whittaker 2018). Diese geben Auskunft über die alltagsübliche professionelle Entscheidungspraxis und haben daher als Blaupause für die Untersuchung der Fallarbeit in der Pandemie eine hohe Relevanz.

Ethnographische Studien fokussieren vor allem das „Wie“ der Urteilsbildung, denn Fachkräfte treffen ihre Entscheidungen nicht im luftleeren Raum, sondern sind eingebunden in ein Netzwerk verschiedener Akteur*innen (Bastian 2017), in dem unterschiedliche Anforderungen und Interessen immer wieder ausgehandelt und austariert werden müssen (Kettle 2018). Kinderschutzarbeit, so Bode (2017), ist netzwerkförmig organisiert und folgt einer *begrenzten Rationalität* (Gigerenzer und Goldstein 1996). Von diesen Überlegungen ausgehend, lässt sich die Arbeit der Fachkräfte im Kinderschutz im Sinnes eines „*muddling through*“ (Bode und Turba 2014), als eine Bearbeitung von Komplexität begreifen.

A. ***Die Rolle des Teams:*** Eine wichtige Bedingung für die Urteilsbildung und Entscheidungsfindung in der Sozialen Arbeit bildet das Team und dessen strukturierende sowie strukturherstellende Funktion. Dies betrifft beispielsweise die Bearbeitung der Arbeitsanforderungen und die Entwicklung eines robusten Rollenverständnisses (Cook 2019). Zudem ist das Team die zentrale Instanz der kooperativen Konstruktionen einer kohärenten Begründungsrationalität (Leigh 2017; Smith 2014; Whittaker 2018). Die Beziehungsaspekte und Teamkulturen sind hinsichtlich der Herstellung fachlicher Urteile von großer Relevanz in der relationalen Einbettung von Bewertungsprozessen bezüglich der Adressat*innen und erzeugen habitualisierte Routinen als Antwort auf die Kontingenz der Aufgaben (Broadhurst et al. 2010). Dieser Konstruktionsprozess hat zudem für die Teammitglieder die Funktion, sich sowohl in offiziellen Teamsituationen (Saltiel 2015) als auch in kollegialen Tür-und-Angel-Gesprächen intersubjektiv zu vergewissern und so eine kollektive Verantwortungsübernahme herzustellen (Helm 2017; White 2002). Professioneller Konsens ist für weitreichende Entscheidungen, wie etwa die Herausnahme eines Kindes aus der Familie, von zentraler Bedeutung (Chateauneuf et al. 2020). Cook und Zschomler (2020) konnten bereits feststellen, dass viele dieser Teamfunktionen in der Pandemie nur teilweise durch digitale Möglichkeiten aufrechterhalten werden konnten (vgl. auch Cook et al. 2020).
B. Fachkräfte nutzen in der Fallarbeit ein **„Sense-Making“** (Doherty 2016; Whittaker 2018), durch das jene die verschiedenen situativ häufig wechselnden Anforderungen, Erwartungen und Handlungsparadoxien stets neu zu einem nach außen und innen vertretbaren, „darstellbaren“ (Ackermann 2017) Sinnganzen zusammenfügen bzw. narrativ herstellen (Bastian et al. 2017). Hierbei spielt auch die Kombination aus intuitiver Heuristik und analytischem Denken eine Rolle (Bastian und Schrödter 2015; Hackett und Taylor 2013; Nyathi 2018). Gerade ethnographische Studien legen dar, wie stark intuitive Heuristiken mit der Logik solcher analytischen Instrumente ausgehandelt werden müssen (Bastian 2017) und, dass die Fachkräfte diese sogar manipulativ verändern oder aber unwissentlich falsch benutzen (Gillingham et al. 2017; Gillingham und Humphreys 2010; Lyle und Graham 2000).
C. In Bezug auf die Interaktion mit Adressat*innen nutzen Fachkräfte implizite ***Testverfahren*** (Freres et al. 2019), indem sie den Familien systematisch „Druck machen“ (Ackermann 2017; Pomey 2017; Retkowski und Schäuble 2011; Schone 2017), oder prüfen, inwiefern Eltern in der Lage sind, sich als ‚normal‘, d. h. hier: als ‚gute‘, angemessen agierende Eltern zu inszenieren.

Die bisherige Forschung zum konkreten Vollzug professioneller Urteils- und Entscheidungsfindung im Kinderschutz gibt wichtige Einblicke in die Handlungsgewohnheiten und impliziten, aber auch expliziten Umgangsweisen, durch die die Fachkräfte in dem komplexen Netzwerk, in dem die Fallarbeit eingebettet und verwoben ist, die dort inhärenten widersprüchlichen Aufgaben und komplexen Problemstellungen bearbeiten. Gleichzeitig zeigen die aufgeführten Studien, wie sich aber die dazu nötigen Rahmenbedingungen durch die Pandemie und die politischen Maßnahmen verändert haben. Es lassen sich zudem erste Hinweise zu veränderten Umgangsweisen der Fachkräfte finden. Dennoch bleibt bisher ein Wissensdefizit darüber bestehen, wie sich diese Umgangsweisen gestalten, etwa in Bezug auf die Krise und die Entwicklung (kreativer) Lösungen im konkreten Vollzug der Fallarbeit, vor allem hinsichtlich der im Forschungsstand aufgezeigten impliziten Strategien in der Arbeit mit den Familien und der Rolle der Teamarbeit.

## Hintergrund, Methoden und Untersuchungsdesign

Grundlage der Studie bilden zwölf Expert*inneninterviews (Meuser und Nagel 2009) mit Fachkräften öffentlicher und freier Träger. Teilweise fanden diese noch während des ersten Lockdowns statt. Die Interviews wurden aufgrund der Pandemie telefonisch durchgeführt und auch die Akquise, Vorbesprechung und das Einverständnis erfolgte über E‑Mail und Telefon. Es wurden vor allem Erzählanreize zur momentanen Situation bzgl. politischer Regularien, zur organisationalen Ebene (Infektionsschutz der Fachkräfte, Unterstützung durch die Leitung etc.) und zur konkreten Fallarbeit während der Lockdownphasen gegeben. Zudem wurden die Fachkräfte zu einer Einschätzung bezüglich der Situation ihrer Adressat*innen gebeten. Ein besonderer Schwerpunkt lag auf Fragen der Zusammenarbeit mit Kolleg*innen sowie der technischen Ausstattung bzw. der Verwendung digitaler Kommunikationsmittel in der Fallarbeit. Die Interviewdauer changierte zwischen etwa 20 und 60 min. Die Basis der hier präsentierten Ergebnisse bildet die Auswertung von vier Interviews mit fallführenden Jugendamtsmitarbeiter*innen und Leitungskräften zweier Großstädte aus unterschiedlichen Bundesländern (ASD A und ASD B). Es handelt sich dabei um eine Leitungskraft eines Bezirkes (ASD A 2), eine Leitungskraft eines Kinderschutzteams (ASD A 5) sowie um zwei ASD-Fachkräfte (ASD B 1, ASD B 2). Diese wurden ausgewählt, da sie die hier beschriebenen Umgangsweisen mit den als Krisen beschriebenen Situationen, die auch in den anderen Interviews von Bedeutung waren, am prägnantesten darlegen. Dennoch bleibt kritisch anzumerken, dass die vorgenommene Auswahl die Aussagekraft der Datenanalyse begrenzt. Es handelt sich somit um eine Studie mit explorativem Charakter, die – da teilnehmende Beobachtungen pandemiebedingt nicht möglich waren – der ethnografischen Haltung der Autor*innen nicht vollumfänglich gerecht wird und doch hoch relevante empirische Hinweise für eine intensivere Debatte zu den Auswirkungen der Pandemie auf die konkrete Praxis in der ASD-Arbeit bietet. Die Studie erweitert daher bisherige breiter angelegte, quantitative Studien (Buschle und Meyer 2020; Mairhofer et al. 2020) durch die hier eingenommene ‚Mikroperspektive‘.

In den Interviews wurde auf Grundlage erzählgenerierender Fragen nach den Veränderungen im Arbeitsalltag, vor allem bezogen auf die konkrete Fallarbeit und Organisation sowie auf den Einsatz neuer technischer Ressourcen und deren Auswirkungen auf die Urteilspraxis, gefragt. Die Auswertung erfolgte auf der Grundlage der Grounded Theory. Durch den ständigen Wechsel zwischen Datenerhebung und Datenauswertung wurde einem wichtigen Prinzip der Grounded Theory nachgegangen. Allerdings war die Auswahl an Interviewpartner*innen durch die Pandemie begrenzt: Nicht wenige Anfragen verliefen mit Verweis auf die hohe Arbeitsbelastung ins Leere, wobei versucht wurde, bereits kurz nach den ersten Lockdowns sehr schnell mit der Studie zu beginnen. Dennoch wurde ein theoretisches Sampling durchgeführt, indem die bereits auf Vorrat gewonnenen Daten auf der Grundlage der sich herausbildenden Schlüsselkategorien durch Strategien minimalen oder maximalen Vergleichens immer wieder mit einbezogen werden konnten (Strübing 2021). Die Datenanalyse erfolgte nach den üblichen Kodierschritten, wie sie Strauss und Corbin (1996) vorschlagen. Zunächst wurden die Interviews im Team in spezifische Abschnitte zerlegt (offenes Kodieren). Dort kristallisierten sich bereits einige Konzepte heraus, von welchen in diesem Artikel insbesondere das der *Krise* von Belang ist (Kap. 4). Im Rahmen des axialen Kodierens wurde durch kontrastierende Vergleiche das Konzept der Krise geschärft und anhand einiger besonders prägnanter Textstellen durch ‚line-by-line-Analysen‘ (Strauss und Corbin 1996) weiter ausgearbeitet und theoretisch kontextualisiert. Dabei verschob sich der Fokus der Analyse mehr und mehr auf die Kernkategorie Umgang der als Krisen erlebten Handlungseinschränkungen (Kap. 5) und den damit verbundenen Unterkategorien „Austausch im Team und Ausgestaltung einer Peripherie“ (Abschn. 5.1) sowie „Nähe und Distanz zu den Eltern konstruieren“ (Abschn. 5.2). Im nächsten Kapitel soll daher zunächst der empirisch gewonnene Krisenbegriff mit Hilfe des von Latour herausgearbeiteten Begriffs der *Gewohnheit* diskutiert werden, um dann auf dieser Grundlage die Ergebnisse hinsichtlich des Umgangs mit der Krise darzulegen.

## Das Wegbrechen gewohnter Handlungsweisen – Krise als Netzwerk

Bei der Untersuchung sozialpädagogischer Interventionen wird der Fachkraft als Akteur*in üblicherweise ein starker Subjektstatus zugewiesen, d. h. es werden hohe normative, professionstheoretisch angereicherte Erwartungen an das professionelle Handeln der Fachkräfte angelegt und das untersuchte Handeln an diesen bemessen (Bastian 2019; Nadai 2012). Kontextbedingungen werden dann eher als flankierende, manchmal auch störende oder das reine professionelle Handeln kontaminierende Einflüsse gewertet. Mit einem relationalen Blick, wie ihn etwa Latour (2017) ausformuliert, lässt sich eine Perspektive auf professionelles Intervenieren entwickeln, welche nicht diese strenge Trennung zwischen Akteur*in und Kontext vollzieht, sondern professionelles Handeln als einen Prozess versteht, der sich durch Verflechtungen und Verbindungen bzw. durch ein wechselseitiges Zusammenspiel verschiedener menschlicher Akteur*innen und nichtmenschlicher Entitäten versteht. Verständlich wird das Handeln der Fachkräfte erst, wenn man es im Kontext betrachtet. Der durch Latours Akteur-Netzwerk-Theorie geschärfte Blick offenbart dann, dass im Grunde alles Kontext ist, da sich kein einzelnes Subjekt ausmachen lässt, das durch andere Entitäten ‚kontextualisiert‘ wird. Die ‚Co-Produktion‘ von Eltern, Kindern und Sozialarbeiter*innen (Müller 2017) sowie Berichten Dritter, von Akten und Dokumenten, institutionellen Zusammenhängen, aber auch von den Erzählungen und Verhaltensweisen der Adressat*innen sind demnach nicht nur der Rahmen, innerhalb dessen *etwas* passiert, sondern diese Dinge sind in ihrer Vermischung *das* was passiert, also das, was die Situation konstituiert.

Seit März 2020 betritt ein neuer sehr einflussreicher Akteur die Bühne der Kinder- und Jugendhilfe, nämlich SARS-CoV‑2. Die Veränderungen, welche das Virus und die darauf bezogenen Schutzmaßnahmen mit sich bringen, werden von den Fachkräften ausführlich thematisiert und zum Teil als sehr bedeutsam erlebt. Viele dieser Veränderungen betreffen den Wegfall von Institutionen, die bislang eine wichtige Rolle im Melder*innennetzwerk eingenommen haben und nun selbst durch Schließungen keinen Kontakt zu potenziellen Adressat*innen hatten. Wie sehr die Allgemeinen Sozialen Dienste auf dieses Netzwerk angewiesen sind, zeigt auch die bereits erwähnte DJI-Studie, in der von einer Stagnation oder sogar von einem Sinken der Meldungen gesprochen wird und dies auf den Wegfall wichtiger Institutionen zurückgeführt wird (Mairhofer et al. 2020).

„[…] wir haben im Normalfall also zu normalen Zeiten wir sind im zwölften Jahr mit diesem Team immer so circa fünfzig Meldungen im Monat (…) im Moment in den ersten Wochen von Corona also im März waren es einundzwanzig also(?) es war erschreckend wenig ähm viel weniger auch Polizeieinsätze wegen häuslicher Gewalt ähm was auch Polizei ja bestätigt hat auch deren Einsatz ähm Volumen hat sich sehr runtergefahren erstmal in der Zeit“ (ASD A 5, Z. 23–29)

In dieser Aussage einer ASD-Leitungskraft im Kinderschutzfachdienst des ASD A aus unserem Sample werden Auswirkungen auf den gewohnten Arbeitsalltag im Kontrast zum „Normalfall“ beschrieben. Dass diese Veränderungen etwas *Krisenhaftes* implizieren, zeigt der Verweis auf „erschreckend“ wenige Fälle von häuslicher Gewalt und der damit verbundene Subtext einer zu hohen Dunkelziffer von nicht erkannten Fällen. Solche Aussagen in den Interviews wurden im Zuge des axialen Codierens zu einer Schlüsselkategorie „Krise“ (Kap. 4) bzw. daran anschließend „Umgang mit Krise“ verdichtet (siehe Kap. 5). Der Begriff der Krise wurde dabei nicht leichtfertig verwendet. Zunächst erscheint die Terminologie zu unpräzise, primär durch seine vermehrte Nutzung im Zuge der Coronapandemie. Ein solcher Krisenbegriff entwirft ein „Panorama“ und verstellt als pure Referenz, Kameraeinstellung oder Assemblage (Latour 2017) durch seine Totalität den Blick auf das eigentlich Krisenhafte bzw. macht dessen Bearbeitung unmöglich: „Sie [die Totalisierung] macht ohnmächtig angesichts des Feindes, weil sie ihn mit phantastischen Eigenschaften ausstattet“ (Latour 2008, S. 166). Die Krise ist nicht ‚die Viruspandemie‘ und auch nicht ‚die Politik‘, diese sind es auch nicht, die das Krisenhafte ‚erklären‘. Vielmehr verteilt sich die Krise in einem Netzwerk, in dem viele unterschiedliche Akteur*innen am Werk sind und die angestoßenen Transformationsprozesse weitergeben, übersetzen und verteilen. Viele dieser konkreten Krisenmomente wurden uns von den unterschiedlichen Fach- und Leitungskräften geschildert. Ihnen gemeinsam ist, dass sie sich durch das sehr plötzliche Wegbrechen *gewohnter* Handlungsweisen auszeichnen – denn dadurch werden sie zu Krisen –, wie das eine weitere Fachkraft eindrücklich schildert:

„Und in den Schutzkonzepten waren dann Schulen miteingeschlossen, es waren Fachkräfte von ambulanten Diensten miteingeschlossen, es waren Beratungsstellen miteingeschlossen. Und es waren Vereine miteingeschlossen. Und das ist dann halt mit einem Schlag weggefallen.“ (ASD B 2, Z. 183–185)

Durch das „mit einem Schlag“-Wegfallen ist der gewohnte Handlungsmodus nicht mehr haltbar. Die *Gewohnheit*, für Latour ein zentraler Existenzmodus, stellt eine Kontinuität dadurch her, dass sie es schafft, Diskontinuitäten zu überwinden und dadurch relativ stabile und dauerhafte Handlungsmodi zu etablieren. Beispiele dazu lassen sich aus dem Forschungsstand heraus bereits erkennen, etwa in dem komplexen Vorgang des Sense-Making, in dem standardisierte, technisierte und zugleich interpretative und erfahrungsbasierte Wissens- und Informationsbeschaffungsformen mühelos in Verbindung gebracht werden oder in der ebenfalls komplexen Zusammenarbeit im Team. Gerade solche Gewohnheiten wurden durch die Pandemie gestört und die Akteur*innen im Netzwerk müssen neu verteilt werden. Latour nutzt für solche Ereignisse den Begriff der *Überraschung* als den Moment, in dem man sich „bei der kleinsten Krise, der kleinsten Kontroverse, der kleinsten Panne einem neuen unvorhergesehenen Element (…) [gegenübersieht], das man der Liste hinzufügen muß [sic!] und an das weder der eine noch die andere gedacht hatten“ (Latour 2014, S. 74) – wodurch sich das Netzwerk verkompliziert und erweitert.

Dieser Prozess der Erweiterung oder Veränderung des Netzwerks lässt sich auch als *Bewältigung* verstehen. Wir haben uns von diesem Begriff allerdings im Laufe der Analyse mehr und mehr verabschiedet, da dieser zu subjektzentriert ist und deshalb die herausgearbeiteten Prozesse offener als „Umgang mit Krise“ bezeichnet. In Latours Perspektive geht es nicht um die Bewältigungsstrategie eines einzelnen Subjekts, sondern um eine Umverteilung und Neuverknüpfung des Netzwerkes vieler Akteur*innen (auch wenn wir durch die Interviewmethode dies leider aus der einen Perspektive des*der Interviewten rekonstruieren müssen). Es ist diese unterschwellige Achtsamkeit, die der Gewohnheit laut Latour eigen ist, die solche Prozesse möglich macht, indem auch im gewohnten Handlungsfluss spontan auf Veränderungen eingegangen werden kann, Diskontinuitäten also nicht vergessen werden, sondern „unter der *vergeßlichen* [sic!] und *reflexhaften* Gewohnheit, *etwas gewacht hat*“ (Latour 2014, S. 376). Damit unterscheidet sich Latours relationales Konzept auch vom strukturalistischen Krise-Routine-Dualismus (Oevermann 2016), da hier ohne Bezugnahme auf Fallstrukturen das verteilte Handeln von verbundenen Akteur*innen ursächlich für das Bewältigungshandeln ist. Routine ist bei Latour daher eine misslungene Gewohnheit, die in Stumpfheit und Automatismus mündet, statt in Aufmerksamkeit (Latour 2014, S. 376).

Neben dem Wegfall des Melder*innennetzwerkes werden noch weitere Veränderungen als krisenhaft beschrieben. Dies sind beispielsweise die Priorisierung einzelner besonders vulnerabler Gruppen, wie die Unter-Dreijährigen oder auch die Konzentration auf Kinderschutzfälle, wodurch andere Gruppen und Fallkonstellationen eine Zeit lang aus dem Blick geraten. Gleichzeitig gewinnen neue schützende Dinge, wie Masken oder Handschuhe, an Relevanz. Durch die Verdinglichung der Fallarbeit bzw. der Fallkategorien verdeutlicht sich schön, wie in der Alltagssprache der Unterschied zwischen menschlichen und nicht-menschlichen Akteur*innen verschwimmt. Gleichzeitig tritt die Technik als neue Akteurin auf, wie sich in der später zitierten Beschreibung des Aufbaus der Peripherie für einen Fernzugriff (Abschn. 5) oder aber auch an der Verwendung technischer Hilfsmittel für den Austausch im Team zeigt.

Diese kurzen Einblicke geben Hinweise auf die konkreten Veränderungen, die sich während der untersuchten Zeiträume vollzogen haben. Es zeigen sich Verschiebungen im Netzwerk, ein Wegbrechen alter und der Einzug neuer Akteur*innen, welche die fachliche Arbeit neu vermitteln und übersetzen. Hierdurch ergeben sich konkrete Anforderungen, die womöglich eingespielte Gewohnheiten in Frage stellen und somit Krisen hervorrufen, die bewältigt werden müssen.

## Der Blick auf den Umgang mit Krisen

Ausgehend von dem im vorangegangenen Kapitel empirisch entwickelten und mit Latour kontextualisieren Krisenbegriffs richten wir den Blick auf das Erleben von Kontingenz bzw. Transformation und das damit zusammenhängende Prekär-Werden der fachlichen Arbeit, sprich, der Umgang mit Krisen. Dieser erste Eindruck der vorläufigen Studienergebnisse bezieht sich bislang auf Spotlights einer Praxis, die an verschiedenen Orten, in differierenden organisationalen Zusammenhängen und zu unterschiedlichen Zeiten stattfindet. Es ist davon auszugehen, dass an Ort A, an dem Interviews bereits *während des Lockdowns* Ende April/Anfang Mai 2020 stattfanden, eine sich dort vorgefundene Praxis unterscheidet von Ort B, an welchem erst im Juni und Juli 2020 Interviews geführt wurden, weshalb diese beiden kontrastierenden Zeitpunkte auch mit in die Darstellung einbezogen wurden. Die beiden Unterkategorien „Austausch im Team und Ausgestaltung der Peripherie“ – welche sich stärker auf die Ebene der Fachkräfte, etwa im Austausch untereinander oder im Umgang mit technischer Ausstattung bezieht – sowie „Nähe und Distanz zu den Eltern konstruieren“ – welche stärker die Fachkräfte-Adressat*innen-Ebene in den Blick nimmt –, die wir zur Kernkategorie Umgang mit Krisen zusammengefügt haben, werden nun im Folgenden dargestellt und durch empirische Beispiele veranschaulicht.

### Austausch im Team und Ausgestaltung einer Peripherie

Trotz der relativen Länge einzelner Interviews und den teilweise längeren Erzählanteilen erscheint besonders auffällig, dass Fallarbeit als konkrete Praxis selten eine Rolle spielt. Stattdessen wird an beiden Standorten in der Retrospektive klar, dass mit Beginn des Lockdowns zunächst einmal – zumindest für eine gewisse Zeitspanne – fast die komplette Arbeit des ASD zum Erliegen kam (kein Publikumsverkehr, Homeoffice) und daher unter den Fach- und Leitungskräften eine große Unsicherheit und eine abwartende Haltung vorherrschte. Danach wurden zunächst einmal als relevant markierte Bereiche (hier vor allem die Verdachtsabklärung im Kinderschutz) re-installiert und eine unterschiedlich lange Übergangszeit zu einer irgendwie funktionierenden Umsetzung des Homeoffice begann. Auf die Einstiegsfrage nach dem eigenen Empfinden antwortet eine Fachkraft aus ASD B:

„Ach mir geht es jetzt inzwischen ganz gut. Wir hatten gestern unseren Teamtag, wo wir das tatsächlich ausführlich nochmal besprochen hatten. Es ging … los im Mitte März, da war das Ganze noch etwas anders (unv.), da hätte ich wahrscheinlich nicht gut gesagt (lacht). Ähm deswegen einfach, dass einfach niemand Erfahrung mit einer Pandemie hat, niemand weiß was zu tun ist und was einen erwartet. Es sind viele Informationen eingegangen von verschiedenen Stellen, was alles zu beachten ist, und da war schon die Überforderung vorrangig (lacht). Ähm in der Zwischenzeit, als sich dann alles eingependelt hat, das mit dem Homeoffice besser geklappt hat, man da irgendwie versucht hat Routinen zu entwickeln, hat das dann doch besser geklappt. Und wir in der Stadt [Name der Stadt] sind ja jetzt seit Anfang der Woche wieder im in Anführungszeichen Normalbetrieb aber mit eingeschränktem Bürgerkontakt, aber es kommt nach und nach wieder die Normalität kehrt wieder ein.“ (ASD B 1, Z. 7–19)

In diesem im Juni 2020 geführten Interview wird explizit ein Prozess des Umgangs mit einer Krise beschrieben. Innerhalb der Sequenz „mir geht es inzwischen wieder gut“ ist der gegenwärtige Endpunkt einer sich ins Positive transformierten Entwicklung zu sehen, an deren Anfang eine Krise gekennzeichnet durch das Wegbrechen der eingeschliffenen Gewohnheiten („niemand weiß, was zu tun ist“) und das angesprochene Überforderungsgefühl stand. Das formulierte Ziel der Normalität wird als neue transformierte „Normalität“, als das „alles einpendeln“ im Sinne eines Funktionierens des Homeoffice und als „Normalbetrieb“ im Zeichen „eingeschränkten Bürgerkontakts“ gefasst. Entscheidend für die interviewte Person ist aber das Team, welches hier die eigentliche Funktion der „Krisenbewältigung“ übernimmt. Der Teamtag und seine kathartische Wirkung, alles noch einmal zu besprechen, verändert sich in seiner grundlegenden Funktion eines Ortes der Reflexion und des fachlichen Austauschs hin zu einer Art *Selbsthilfegruppe* zum Umgang mit der Krise (oder war in diesem Fall schon immer ein solcher Ort, z. B. als kollegiale Beratung). Die interviewte Person geht jedenfalls vollkommen in diesem Team auf, denn die Frage nach ihrem eigenen persönlichen Empfinden wird im zweiten Satz sogleich kollektiviert und das „Ich“ durch ein „Wir“ ersetzt. Das Team und dessen strukturierende und strukturherstellende Funktion ist ein in den Interviews immer wieder aktualisiertes Motiv, auf dessen Bedeutung für bestimmte Gewohnheiten etwa des kooperativen „*Sense-Making*“ im Forschungsstand bereits hingewiesen wurde. Doch hier zeigt sich eine Teamfunktion, die nicht auf die Fallarbeit bezogen wird, sondern die das Team als Zusammenschluss der von der Krise Betroffenen zur gemeinsamen Bewältigung darstellt. Die Krise wird als eine Überforderung, zugleich jedoch auch als Paralyse, in Form des Kontaktverbotes mit Adressat*innen und der Verbannung aus den Büros ins häusliche Umfeld, ohne dass dafür eine technische Infrastruktur zur Verfügung stand, erlebt. In einem Interview mit einer Leitungskraft von Mai 2020, also inmitten des Lockdowns, wird dieser Prozess erläutert:

„Das Rathaus ist jetzt schon seit ’ner längeren Zeit gesperrt für die Bürger, da gibt es nur bestimmte Ausnahmesituationen sag ich mal, wo dann direkter Face-to-Face-Kontakt stattfinden kann. Und das ist schon eine wesentliche Einschränkung. […]. Also ich jetzt auch das Glück, dass ich sag ich mal relativ abgeschottet im Dachgeschoss sitzen kann und da auch mittlerweile einen Fernzugriff hab auf das ganze System und auch auf Fachverfahren, das war am Anfang auch nicht so. Ähm also in den ersten Tagen des Homeoffices war es so, dass ich ein Smart /, ein Dienstsmartphone hatte mit einer E‑Mail-App und ähm ich hab mich dann selber ein bisschen schlau gemacht und hab mir so ein USB-Adapter gekauft, damit ich meine Computertastatur an das Diensthandy anschließen kann, damit ich darüber meine E‑Mails tippen kann und nicht, ne, quasi auf dem /, auf dem Display vom Handy rumdrücken muss. Und dann so peu á peu wurde ein Fernzugriff geschaltet, dass ich dann sozusagen mit Bereitstellung meiner privaten Peripherie, also privater Laptop bereitstellen, dann ein Fernzugriff auch hatte auf das ganze System und dann konnte ich nahezu uneingeschränkt von Zuhause aus auch arbeiten. Also da hatte ich auch nochmal Zugriff auf E‑Mails, Fachverfahren, auf die ganzen städtischen Laufwerke und so weiter und das war dann auch eine echte Erleichterung und ich finde das gibt auch einen Ausblick so auf die Perspektive, Möglichkeiten zu nutzen von Zuhause aus zu arbeiten.“ (ASD A 2, Z 138–147)

Hier werden die Schließung und die erlebte Einschränkung des *Face-to-Face-Kontaktes* angesprochen, was zunächst einmal als eine „echte Einschränkung“ wahrgenommen wurde. Interessant ist jedoch die Wendung im nächsten Satz, in der nicht etwa die Aufhebung dieser Einschränkung gewünscht wird, sondern die Abschottung im privaten Dachgeschoss als *Glück* erlebt wird. Danach wird das Augenmerk komplett auf die Frage des „Fernzugriffs“ gelenkt, also der Möglichkeit, mithilfe technischer Peripherie aus der Abschottung heraus *aus der Ferne* den Zugriff auf etwas zu erlangen, das nicht näher expliziert wird. Auch spätere Verweise auf die Möglichkeiten „irgendwie auf’s Fachverfahren zurückzugreifen oder in die Einwohnermeldedaten zu gucken oder mal ’ne Mail zu schreiben“ bleiben recht uneindeutig und es werden keine Bezüge zur eigentlichen Fallarbeit hergestellt. Wichtiger erscheint der persönliche Einsatz, mit dem die Person sich die Technik unter Rückgriff auf private Ressourcen nutzbar macht, eine Tätigkeit, die ihre komplette Aufmerksamkeit in Anspruch zu nehmen und für die betreffende Person eine Selbstwirksamkeitserfahrung darzustellen scheint sowie den erlebten Einschränkungen, die die eigentliche Fallarbeit verunmöglichen, gegenübersteht.

Interessant ist in diesem Fall auch die Fragestellung im Interview, denn es wurde eben nicht nach dem Prozess der Schließung, sondern nach dem neuen Arbeitsalltag und nach expliziten Fallgeschichten gefragt. Diese kommen aber nicht vor, auch nicht an anderer Stelle im Interview. Dies deckt sich mit dem Interview *ASD B 1*, nur dass bei *ASD A 2* die Technik als Surrogat für die „Lücke der Fallarbeit“ und im ersten Fall das Team als „Selbsthilfegruppe“ diese Leerstelle ausfüllt. In beiden Fällen wird der Verlust der regulären Fallarbeit als krisenhaft erlebt – denn – so die These – dadurch fiel zusätzlich ein Stück Selbstvergewisserung weg. Dementsprechend war teilweise die genaue Vorgehensweise unklar, bzw. der eigentliche Inhalt des Jobs missverständlich. In Bezug auf die zuvor beschriebene Selbstwirksamkeitserfahrung handelt es sich auch um eine Vergewisserung der eigenen Handlungsfähigkeit, welche sich zwar nicht (mehr) in einer erfolgreichen Fallarbeit aber zumindest in der Beherrschung der Technik äußert.

Die Verwiesenheit in das Homeoffice, ohne eine Peripherie, wirft die Fachkräfte zum Teil in eine gezwungene Untätigkeit, die bewältigt werden muss. Dieses krisenhafte Aufbrechen von Handlungsgewohnheiten zeigt sich mehrfach durch den Ausdruck des „Reinschmeißens“ („in das kalte Wasser geschmissen“ (*ASD B 1*); „weil wir natürlich ich sag jetzt mal ins Homeoffice geschmissen worden sind“ (*ASD A 5*)) bei einer gleichzeitigen Entgrenzung des privaten und beruflichen Bereichs (Nutzung von Heimcomputern, Privathandy oder die Nutzung von Diensthandys außerhalb der Dienstzeit). Während in B1 ein Leidensweg, ein Aushalten bis zum befreienden Teamtag geschildert wird, nutzt die Person *ASD A 2* das „Herumhantieren“ mit der Technik, die nach und nach immer mehr Möglichkeiten bis hin zu einem Fernzugriff gewährt als Form des Umgangs mit Krise und hybridisiert dadurch den privaten und den beruflichen Raum und sein fachliches Handeln (Freres et al. 2019) durch die Ausgestaltung einer Peripherie. Anders als *ASD B 1*, die erst wieder den Normalbetrieb als Rettung sieht, findet sich *ASD A 2* in seiner neuen Situation der Abschottung, in Anwesenheit der Peripherie, zurecht und erlebt dies als „echte Erleichterung“, die so weitergeführt werden könnte.

Konträr hierzu zeigt sich Interview *ASD B 2*, welches einige Zeit nach den Öffnungen geführt wurde. Hier wird, wie auch in *ASD B 1, *auf das Team und dessen Funktion verwiesen. Zeitgleich wird die Reaktion auf den Lockdown jedoch als ein strukturiertes und schnelles Vorgehen beschrieben:

„Also ich habe mit zwei Wochen Homeoffice gestartet, nachdem die Ausgangsbeschränkungen in Kraft getreten sind. Ich hatte gleich als erstes mit meinem Homeoffice Zugang. Das war ganz gut, weil ich zumindest Mails beantworten konnte. Und dann war ich zwei Wochen im Amt eingesetzt. Dann war ich zwei Wochen wieder im Homeoffice und dann hatten wir so eine Regelung, dass man teilweise, also tageweise, dort war.“ (ASD B 2, Z. 20–25)

In dieser Erzählung reißt der Kontakt zu den Fällen niemals ab und so sind auch an späteren Stellen immer wieder Erzählungen darüber sichtbar, wie folgendes Beispiel verdeutlicht:

„Also jetzt mal ein ganz praktisches Beispiel. Ich habe ein Kind in Obhut genommen. Da war die Polizei dann auch mit dabei und wir hatten zum Glück zehn Schutzmasken für uns, weil wir natürlich in den Haushalt mussten. […] Und dann hatten wir eben das Kind, das heißt wir waren zu dritt. Dann hatten wir das Problem, dass man im Taxi nur hinten sitzen darf, wir dann halt zu dritt ganz eng mit dem Kind im Taxi waren und es in der Klinik die Einschränkung gab, es darf nur eine Person mit dem Kind rein zur Untersuchung. […] Also von daher Inobhutnahme war schon möglich, aber das hat es halt alles sehr erschwert.“ (ASD B 2, 43–54)

Der Verweis auf das „ganz praktische Beispiel“ kommt bereits sehr früh im Interview und somit zeigt die Erzählung der Fachkraft eine eigentlich typische oder zumindest zu erwartende Form eines Interviews mit einer Expert*in, da hier über die eigentliche Tätigkeit, den eigentlichen „Job“ gesprochen wird und nicht über Dinge, die eher als Kontexte (das Team, technische Peripherie) in der Kinder- und Jugendhilfe eingeordnet werden. Die Fallbeschreibung geht dabei explizit auf die Auswirkungen der Abstands- und Hygieneregeln ein und setzt diese in Bezug auf die am Standort typischen Bestandteile einer Inobhutnahme (Hausbesuch, Transport, medizinische Abklärung in einer Klinik). Eine Inobhutnahme, und somit die eigentliche Fallarbeit, wird erschwert, aber nicht verunmöglicht. Viel mehr noch ist es bei der Inobhutnahme scheinbar nicht möglich, die geltenden Abstandsregelungen einzuhalten. Die Fachkräfte verzichten hier gänzlich auf diese und räumen somit der Inobhutnahme einen Vorrang gegenüber der Abstandsregelung ein. Dies verweist darauf, dass trotz veränderter Rahmenbedingungen auf die übliche Routine der Inobhutnahme zurückgegriffen wird. Zeitgleich zeigt sich im Kontrast zu den vorangegangenen Interviews eine Zweckgerichtetheit auf eine klare Aufgabenstellung des ASDs (Inobhutnahmen vornehmen) und dessen Herausforderungen in einer Pandemie, während in den beiden anderen Fällen kein expliziter Verweis auf eine solche Aufgabe vorkommt und insofern die Kontextbedingungen als alleinstehend, als eigentliches Zentrum der Tätigkeit und nicht als ein Mittel zum Zweck der ASD-Arbeit, präsentiert werden.

In den anderen Interviews wird die eigentliche Fallarbeit bemerkenswerterweise zu einem „Ort des Schweigens“ (Clarke 2012, S. 123), also einer Position, eines Themas, das in einem Interview zur Fallarbeit in den Zeiten einer Pandemie zu erwarten gewesen wäre, jedoch nicht auftaucht, obwohl alle vier Befragten Fall- bzw. Führungsverantwortung haben und in den Interviews auch explizit zur Fallarbeit während der Pandemie befragt wurden. Diese Nicht-Thematisierung lässt sich auch mit Blick auf den Forschungsstand kontextualisieren. Die zuvor beschriebenen üblichen Strategien des Sense-Making und der Testverfahren, die ja die relevantesten *Gewohnheiten* (im Sinne Latours) darstellen, durch welche die Komplexität der Fallarbeit reduziert wird, sind auf die direkte Interaktion mit den Adressat*innen angewiesen. Da diese Interaktion nur sehr eingeschränkt im Lockdown stattfand und auch kaum durch andere z. B. technische Mittel kompensiert wurde, wurden diese gewohnten Handlungsweisen zumindest für diesen Zeitabschnitt verunmöglicht.

### Nähe und Distanz zu den Eltern konstruieren

Ein weiterer Aspekt, der womöglich als Muster des Umgangs mit der Krise gefasst werden kann, zeigt sich in der Adressat*innenkonstruktion. Auch wenn im ersten Muster die Fallarbeit als konstitutiver Teil professioneller ASD-Arbeit unsichtbar blieb, wird dennoch auf eine diffuse Gruppe an Adressat*innen verwiesen. Gleichzeitig wird aber die Situation dieser Adressat*innen immer wieder mit der der Fachkräfte selbst in Bezug gesetzt. Obwohl die Bearbeitung von und der Umgang mit Krisen als Aufgabe von Sozialer Arbeit per se bezeichnet werden kann (Oevermann 2009), ist die Pandemie eine allgegenwärtige Krise, die zunächst einmal alle in ihrer je eigenen Lebenspraxis betrifft. Die von uns gestellte Eingangsfrage zielte auf das eigene Befinden und wurde in einigen Interviews auch zunächst einmal persönlich mit Verweis auf die eigenen Familien, wegfallende Betreuungsangebote und der Herausforderung des Homeschoolings beantwortet. Gleichzeitig führt eine solche Selbstbezüglichkeit des Krisenerlebens in den Interviews beinahe zwangsläufig zu einer Abgrenzung des eigenen lebensweltlichen Handelns von dem der Adressat*innen.

„Aber man fühlt sich, oder ich fühl mich an sich relativ gut informiert. Ich geh davon aus, dass das die [Name der Stadt] Bürger auch sind. Aber gerade in den ersten Wochen gab es auch vermehrt Anrufe von Patchwork-Familien, von getrennt-lebenden Eltern, die trotz sag ich mal der guten Information, dass z. B. Umgangsregelungen nicht von der Kontaktsperre betroffen sind, trotzdem immer nochmal, ne, auch angerufen haben und nachgefragt haben, wie ist das denn und darf ich denn meine Tochter besuchen und die mit nach Hause nehmen oder ist das verboten und was droht mir. Also trotz der eigentlich guten Informationsquellen, wird ja sag ich mal breitgetreten in den Medien, ähm sind Bürger teilweise trotzdem immer noch auch verunsichert.“ (ASD A 2, Z. 28–37)

Die interviewte Person thematisiert an dieser Stelle die eigene Informiertheit bezüglich der Krise zu einem noch frühen Zeitpunkt des *Lockdowns*. Gleichzeitig changiert die Erzählung hier zwischen dem eigenen persönlichen Wissensstand und der allgemeinen Kenntnisse der „Bürger“, die aus Sicht der Person *gut informiert* sind. Der Verweis auf den „gut informierten Bürger“ ist interessant, da damit ein Typus aktualisiert wird, den Alfred Schütz in seinem Essay von der Distribution des Wissens als einen guten Bürger darstellt, der versucht „vernünftig begründete Meinungen auf dem Gebiet zu erlangen, die seinem Wissen entsprechen und ihn zumindest mittelbar angehen, obwohl sie seinem zuhandenen Zweck direkt nicht beitragen“ (Schütz 1972, S. 88). Die interviewte Person zählt sich zu diesen guten „Bürgern“, die – und hier erfolgt eine Abgrenzung – über die „guten“ Informationen verfügen im Gegensatz zu anderen Personengruppen, die als Adressat*innen (Patchwork-Familien, getrennt-lebende Eltern) identifiziert werden könnten. Die Verunsicherung, die sie in den Adressat*innen hervorgerufen sieht, führt der interviewten Fachkraft letztlich vor Augen, dass sie gut informiert ist und in der Lage, aus diesen Mitteilungen Klarheit zu extrahieren, was sie von ihren Adressat*innen unterscheidet.

Dennoch lässt sich die Frage stellen, ob die hier präsentierte Adressat*innenkonstruktion ein im Zuge der Pandemie erfolgtes Umgangsmuster oder die Fortführung gewohnter Kategorisierungen ist. Es lässt sich empirisch zeigen, dass die Konstruktionen von Adressat*innen in der Sozialen Arbeit durch defizitäre Zuschreibungen (Hall und Slembrouck 2011; Messmer und Hitzler 2007) ein essentieller Teil professioneller Tätigkeit darstellt, denn – wie Nina Thieme (2013) ausführt – „[w]ürden […] professionelle Akteure Sozialer Arbeit ihre Kategorisierungen permanent als durch sie Konstruiertes hinterfragen, käme dies einer Negation jener Voraussetzungen gleich, auf deren Basis sie ihr Handeln begründen. Die Konsequenz läge in einer Verunmöglichung des eigenen Handelns“ (Thieme 2013, S. 203). Diese Defizitzuschreibungen im Hinblick auf Normalitätsvorstellungen, wie bereits im Forschungsstand hinsichtlich der Testverfahren diskutiert, die vor allem an das idealtypische Modell des Normalarbeitsverhältnisses und einem damit kongruenten Familienideal orientiert sind (Seelmeyer 2008), bilden nach wie vor eine wichtige Kategorisierungsfolie für Klientifizierungsprozesse. Gleichwohl lässt sich die Defizitzuschreibung auch damit erklären, dass ein erzieherischer Bedarf, also ein Defizit der Eltern vorliegen muss, um Adressat*in der Kinder- und Jugendhilfe zu werden (Schrödter 2020). Die damit einhergehende Abgrenzung der Professionellen von ihren Adressat*innen erlangt aber durch die Pandemie eine neue Bedeutung, da die dadurch erfolgten Einschränkungen (Lockdown, Schul- und Kitaschließungen, Homeschooling) alle oder weite Teile der Bevölkerung betreffen – es ließe sich also zunächst einmal von ganz vergleichbaren Erfahrungen ausgehen, da ja alle im* selben Boot sitzen. *Die Abgrenzungsversuche bezüglich des Umgangs mit Wissen zeugen jedoch davon, dass man eben nicht im selben Boot sitzt. Ähnlich wie am Beispiel der Beherrschung der Technik im vorangegangenen Kapitel dient die starke Unterscheidung hinsichtlich des Informiertheitsstatus jedoch wieder als Akt der Selbstvergewisserung in Zeiten der Unsicherheit und lässt sich in diesem Sinne ebenfalls erneut als Antwort auf die Krise fassen (inwiefern die Transformation von Unsicherheit Teil des „ganz normalen Chaos“ des Jugendamtshandelns ist, zeigen eindrücklich Bode und Turba (2015)).

Das vorangegangene Beispiel ist interessant, da es sich hier um ein Interview handelt, in dem sehr viel Privates berichtet wurde. Zwar wird in einem anderen Interview auch auf das Problem der Herstellung eines strukturierten Alltags verwiesen, allerdings werden im Rahmen dessen strukturelle Gründe genannt:

„Und das andere ist, auch das ähm was ich bei meinen Familien merke, dass gerade einfach wahnsinnig viel verlangt wird von den Eltern ähm zum Thema Hausbeschulung oder auch Kinder, die nicht in den Kindergarten gehen konnten. Die Schulen machen das ja jetzt meistens so, dass sie so Blockunterricht haben. Eine Woche Schule, einen Woche Pause. Und das fordert die Eltern schon sehr. Und gerade Eltern, die sich sowieso ein bisschen schwer tun ihre Kinder zu fördern oder denen so einen strukturierten Alltag zu verschaffen. Da ist es besonders schwer.“ (ASD B 2, Z. 18–26)

Dieser eher relationale Blick, der die individuellen Familien eingebettet in kontextuelle Bedingungen beschreibt, zieht sich durch das Interview – bei einer gleichzeitigen Abwesenheit von Erzählungen über das private Erleben der Krise. Durch die paternalistische Färbung wird von der Fachkraft eine Expert*innenanalyse über die Verfassung „meiner Familien“ angeboten, wodurch simultan eine Nähe konstruiert wird, fast eine Privatheit zwischen der Person und „ihrer“ Familien und eine Distanz über die hier explizit eingenommene Expert*innenrolle, die wiederum die Familien klientifiziert.

## Fazit

Den Ausgangspunkt dieses Beitrags bildete zunächst ein relationaler theoretischer Blickwinkel, wie ihn vor allem Bruno Latour in seiner Sichtweise der Akteur-Netzwerk-Theorie entwickelt, in der das Prozesshafte und kollektiv Vermittelte von Handlungen, Urteilen und Entscheidungen herausgestellt wird und somit der Gegensatz zwischen Situation und Kontext aufgelöst sowie durch einen netzwerkartigen Zugriff ersetzt wird. Dabei haben wir versucht, den Blick weg von der traumatischen Krise „Covid-19-Pandemie“, welche sich durch ihr Hereinbrechen in den Alltag auszeichnet, hin zu den konkreten Entscheidungskrisen im Kinderschutz zu führen, wie sie in den Interviews thematisiert wurden oder sich zumindest aus den Erzählungen rekonstruieren und sich zum Teil bereits in anderen Studien finden lassen.

Auch wenn wir sie in diesem Bericht der Ergebnisdarstellung vorangestellt haben, entwickelten sich diese Perspektiven erst in den empirischen Auswertungen als adäquate Anschlusspunkte, um die gewonnenen Daten besser zu verstehen und im klassischen Sinne einer „*constant comparative method*“ (Glaser und Strauss 1967, S. 101) in ein komparatives Verhältnis zueinander stellen zu können.

Die dargelegten von uns als Umgangsweisen mit Krisen gefassten Aspekte verweisen und stehen in einem engen Verhältnis zu dem Netzwerk, in dem sie sich vollziehen und den Transformationen, die sich zu Beginn und in der ersten Phase der Pandemie vollzogen haben. Insgesamt zeigt sich, dass es zu einer Verschiebung und Neuverteilung der Akteur*innen des Netzwerks gekommen ist, in dem sich einige Akteur*innen, wie etwa die Melder*innengruppen teilweise zurückziehen oder nur noch durch indirekte Verbindungen, wie etwa auf digitalem Wege, auftreten. Dies wiederum hat Auswirkungen, etwa auf den Zugriff auf Fälle, auf das Erleben von Teamarbeit sowie auf das ins Verhältnissetzen der Fachkräfte zu den Eltern. Parallel hierzu sind es die nicht-menschlichen Akteur*innen, die als neue Mittler ins Spiel kommen, sei es als Schutzmaßnahme, in der Gestalt von Masken, Handschuhen oder Desinfektionsmittel oder als technische Akteur*innen, die mit den menschlichen Mitspieler*innen hybride Verbindungen eingehen.

Die aufgezeigten Umgangsweisen sind somit nicht *die* „Bewältigung“ der *Corona-Krise*, sondern lassen sich als Antworten auf *konkrete* Krisen deuten, die auf ein Wegbrechen *konkreter Gewohnheiten* verweisen, wie sie im Abschn. 2.2 exemplarisch aus den Ergebnissen empirischer Studien vorgestellt wurden. Gleichzeitig lässt sich kritisch hinterfragen, inwiefern – auch mit Blick auf den Forschungsstand – Aspekte, wie die besondere Bedeutung des Teams hinsichtlich des eigenen Wohlbefindens oder Abgrenzungspraktiken der Fachkräfte gegenüber den Adressat*innen überhaupt neue Formen des Umgangs mit Krisen darstellen, oder ob nicht permanent Krisenhaftes der professionellen Arbeit durch die benannten Transformationen nun stärker hervortritt.

Es bleiben nicht nur diesbezüglich Fragen offen. Interessant an den Interviews bleibt auch das Nichtgesagte, die Themen, die zwar erwartbar waren, jedoch nicht thematisiert wurden – in dieser Untersuchung die Fallarbeit, die in vielen der Interviews, zum Teil mit großen rhetorischen Mühen, unsichtbar gemacht wurde. Um zu untersuchen, welche Transformationen sich in einer ASD-Praxis im Homeoffice, mit Hilfe neuer digitaler Tools und Möglichkeiten (Schrödter et al. 2020), eingeschränktem Melder*innennetzwerk, ohne oder mit wenig Teamkontakten usw. vollziehen, bedarf es aus unserer Sicht neben Interviews auch teilnehmender Beobachtungen, in denen Praktiken und somit konkrete Prozesse des Umgangs mit Krisen sichtbar werden, welche von den Fachkräften nicht artikuliert werden können. Die hier aufgeführten Ergebnisse bilden demnach nur den Startpunkt einer Forschung, die den Akteur*innen an andere relevante Orte folgt, zu den freien Trägern, den Familien, den Melder*innen und auch zu anderen sichtbaren und unsichtbaren Orten. Die Nichtsichtbarkeit der Fallarbeit im Kinderschutz verweist auf ein Desiderat, oder vielmehr auf einen Forschungszugang, welcher sich durch die Parole „Zurück zu den Fällen“ auf den Punkt bringen lässt. Eine Untersuchung muss die Fälle sichtbar machen, dort wo sie zu finden sind, nämlich in Form von Objekten, wie Akten und Assessmentbögen, die diese Fälle materialisieren, und so als *Formen *weitertransportieren und trans-*formieren *(Latour 2017), so wie Akten eine Transformation der Handlungspraxis der ASD-Fachkräfte in eine fallkompatible Sprache darstellen. Auf der Grundlage solcher Dokumente lässt sich der Blick zurück auf die Fälle wenden und von da auf das Hilfesystem, also das Netzwerk verschiedener Träger, Institutionen, menschlicher Akteur*innen und Objekte richten.
